# Identifying dementia cases with routinely collected health data: A systematic review

**DOI:** 10.1016/j.jalz.2018.02.016

**Published:** 2018-08

**Authors:** Tim Wilkinson, Amanda Ly, Christian Schnier, Kristiina Rannikmäe, Kathryn Bush, Carol Brayne, Terence J. Quinn, Cathie L.M. Sudlow, UK Biobank Neurodegenerative Outcomes Group and Dementias Platform UK

**Affiliations:** a Centre for Clinical Brain Sciences, University of Edinburgh, Edinburgh, Scotland; b Usher Institute of Population Health Sciences and Informatics, Nine Bioquarter, Edinburgh, Scotland; c Institute of Public Health, Cambridge University, Cambridge, UK; d Institute of Cardiovascular and Medical Sciences, University of Glasgow, Glasgow, Scotland; e UK Biobank, Coordinating Centre, Stockport, UK

**Keywords:** Dementia, Alzheimer's disease, Dementia, Vascular, Clinical coding, Epidemiology, Prospective studies, Cohort studies, Sensitivity, Positive predictive value, Predictive value of tests

## Abstract

**Introduction:**

Prospective, population-based studies can be rich resources for dementia research. Follow-up in many such studies is through linkage to routinely collected, coded health-care data sets. We evaluated the accuracy of these data sets for dementia case identification.

**Methods:**

We systematically reviewed the literature for studies comparing dementia coding in routinely collected data sets to any expert-led reference standard. We recorded study characteristics and two accuracy measures—positive predictive value (PPV) and sensitivity.

**Results:**

We identified 27 eligible studies with 25 estimating PPV and eight estimating sensitivity. Study settings and methods varied widely. For all-cause dementia, PPVs ranged from 33%–100%, but 16/27 were >75%. Sensitivities ranged from 21% to 86%. PPVs for Alzheimer's disease (range 57%–100%) were generally higher than those for vascular dementia (range 19%–91%).

**Discussion:**

Linkage to routine health-care data can achieve a high PPV and reasonable sensitivity in certain settings. Given the heterogeneity in accuracy estimates, cohorts should ideally conduct their own setting-specific validation.

## 1 Introduction

The increasing burden of dementia is a cause for major public health concern worldwide [1]. Dementias develop as the result of a complex interplay between genetics, lifestyle, and environmental factors. The effect of any single risk factor is therefore likely to be modest, meaning that very large study populations are required to generate sufficient cases to study associations of exposures with incident dementia. Furthermore, because the pathological processes underlying dementia begin many years before the symptom onset [2], prospective, population-based studies that recruit participants in midlife or earlier will be crucial in understanding natural history and in identifying risk factors and causal exposures.

For prospective, population-based studies to be used for research into the determinants of dementia, participants developing dementia (the “cases” in nested case-control or case-cohort studies) must be identified. One method of doing so is through linkages to routinely collected, coded health-care data sets, which are administrative data sets collected primarily for healthcare purposes, rather than to address specific research questions (e.g., hospital admissions or national mortality data) [3]. Such data sets potentially provide a cost-effective means of identifying disease cases in prospective studies while minimizing loss to follow-up [4].

Participants who develop dementia during follow-up must be identified with a high positive predictive value (PPV); that is, a high proportion of those identified as having dementia in routinely collected data sets should be true dementia cases. Ideally, to maximize statistical power and minimize selection bias in the ascertainment of cases, these sources would also have a high sensitivity, so that a high proportion of all true cases are identified. Specificity and negative predictive values are less relevant metrics, as specificity will be high when precise diagnostic codes are used and negative predictive value, which is related to disease prevalence, will be high in population-based studies where most individuals do not develop the disease of interest.

Hence, a key focus for population-based prospective studies worldwide is to understand the accuracy of dementia codes in routinely collected health-care data sets for identifying dementia cases during follow-up. We therefore sought to systematically identify, evaluate, and summarize all relevant studies of the accuracy of dementia coding within these data sources.

## 2 Methods

### 2.1 Study protocol

We prospectively published the protocol for this review on PROSPERO (www.crd.york.ac.uk/PROSPERO/display_record.asp?ID=CRD42015027232).

### 2.2 Search strategy

We searched the databases MEDLINE (Ovid), EMBASE (Ovid), Web of Science (Thomson Reuters), CENTRAL (Cochrane Library), and PsycINFO (Ovid) for potentially relevant studies published between 1/1/1990 and 14/09/2017. We developed the search strategies with assistance from an information specialist (Supplementary Appendix A). We also identified relevant studies through personal communication and reference list searching.

### 2.3 Study selection

We included studies that compared the presence of codes for dementia and/or its subtypes in any routinely collected health-care data set to any expert-derived reference standard for dementia. We excluded studies that only validated one routinely collected data set against another. Studies had to report either PPV and/or sensitivity or provide data from which either could be calculated. We included relevant studies published in full and as abstracts. We excluded studies that only assessed Creutzfeldt-Jakob disease because it is a notifiable disease in many countries. Where two studies appeared to have overlapping patient populations, we included the study with the largest sample size, and where two different coding systems were investigated separately, we selected the most recent version. We did not impose language restrictions on the search, and translated articles when necessary. We excluded studies with <10 coded events, as we considered these to have limited precision. Studies assessing sensitivity had to be population based (as opposed to hospital or clinic based) and to have made comprehensive attempts to ascertain all dementia cases within that population. We did not impose this restriction on studies reporting PPV because to investigate PPV, the cases are obtained from a routinely collected data set, and the population depends on the data source (for example, for hospital admissions data, all cases will have been admitted to hospital). Two authors (T.W. and A.L. or K.B.) independently screened all abstracts and full-text articles, resolving any discrepancies through discussion and the assistance of a third, senior author (C.L.M.S.).

### 2.4 Data extraction

Two authors (A.L. and T.W.) independently extracted data from the full-text articles of included studies using a pretested standardized template. We extracted information on the following: year of publication; year(s) from which coded data were obtained; country; study population; mean or median age of dementia cases or, if neither was available, the age range of participants at recruitment; study size; the health-care data sets investigated; coding system; coding position; the reference standard to which the routinely collected data sets were compared; and the dementia subtypes (such as Alzheimer's disease [AD] or vascular dementia) investigated. We defined the study size for studies investigating PPV as the total number of participants with a dementia code (i.e., true positives and false positives) and for studies investigating sensitivity as the total number of dementia cases in the population according to the reference standard (i.e., true positives and false negatives). We contacted study authors to obtain key data items that were not reported in publications (e.g., sample size or coding system).

### 2.5 Risk of bias and applicability assessment

We assessed the risk of bias and applicability for included studies using an adapted Quality Assessment of Diagnostic Accuracy Studies 2 form [5]. The Quality Assessment of Diagnostic Accuracy Studies 2 form requires the risk of bias and applicability to be graded (low, unclear, and high) across four categories: patient selection, routine data set used (“index test”), reference standard, and study participant flow (Supplementary Appendix B). Two authors (A.L. and T.W.) independently performed the assessments and resolved discrepancies through consensus. To minimize the risk of study selection bias, we decided to not exclude studies based on the quality ratings (which are inherently subjective), but instead to aid interpretation of results by highlighting those studies, we considered to be at high risk of bias or of applicability concerns.

### 2.6 Data synthesis

We did not perform a meta-analysis given the heterogeneity between study settings and methodologies. Instead, we performed a descriptive analysis of the study results, displaying the range of values in forest plots for visual interpretation. We calculated 95% confidence intervals by the Clopper-Pearson (exact) method. We also reported any relevant within-study analyses that evaluated the effects on PPV or sensitivity of changing a single variable (e.g., selecting people with a dementia code in the primary position compared with those with a code in any position). We performed analyses in R (www.r-project.org).

## 3 Results

### 3.1 Study characteristics

**Fig. 1:**
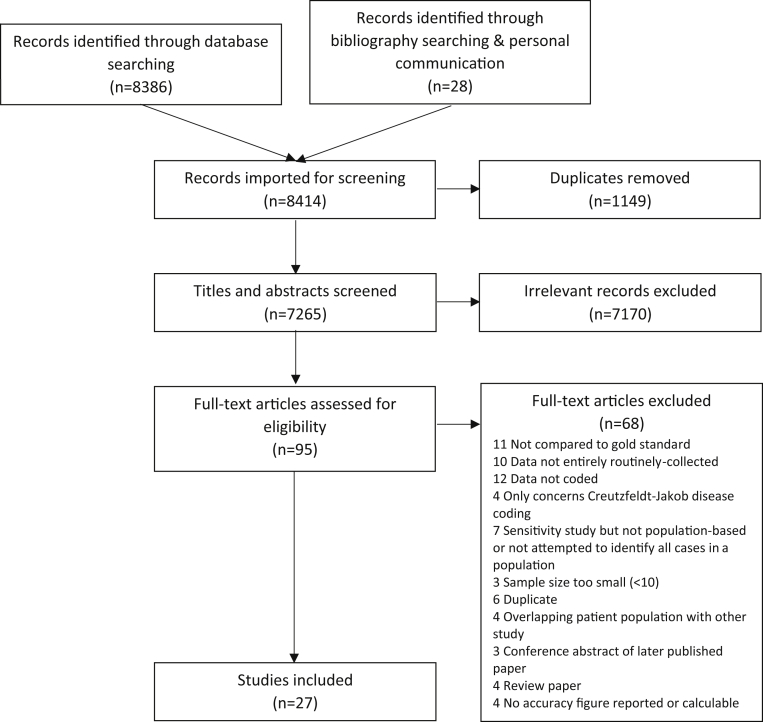
Study selection process.

We included 27 studies [6], [7], [8], [9], [10], [11], [12], [13], [14], [15], [16], [17], [18], [19], [20], [21], [22], [23], [24], [25], [26], [27], [28], [29], [30], [31], [32], of which 26 had full publications [6], [7], [8], [10], [11], [12], [13], [14], [15], [16], [17], [18], [19], [20], [21], [22], [23], [24], [25], [26], [27], [28], [29], [30], [31], [32] and one a published conference abstract [9]. We obtained further details required for analysis from the lead author of the abstract. Fig. 1 outlines the selection process and reasons for study exclusion. Of the 27 included studies, 25 reported PPV [6], [7], [8], [9], [10], [11], [12], [13], [14], [15], [16], [17], [18], [19], [20], [21], [22], [23], [24], [25], [26], [27], [28], [29], [30], and eight reported sensitivity [6], [8], [9], [18], [25], [26], [31], [32] (five reported both). Characteristics of studies reporting PPV and sensitivity estimates are displayed in Tables 1 and 2, respectively.

**Table 1:** Studies reporting the positive predictive value of routinely collected data sets for the ascertainment of dementia cases

First author and publication year	Country	Study period	Study population	Age∗	Study size	Routine data set	Coding system	Code(s) assessed	Coding position	Reference standard and diagnostic criteria if used
Ostbye 1999	Canada	1991–1996	Population based (CSHA)	>65 at recruitment	240	D	ICD 9	331.0, 290.0–290.3, 290.8–290.9, 290.4, 291.2, 291.8, 294.0–294.9, 332.0–332.1, 333.4. 797	Any	Clinical evaluation—DSM III and NINCDS-ADRDA
Bjertness 1998	Norway	1990–1991	Nursing home residents	Mean 85	26	D	ICD 9	290.0, 290.1, 331.0	Any	Clinical and neuropathological diagnosis
Romero 2014	Spain	1993–2007	Population based (NEDICES)	Mean 82	148	D	ICD 9 ICD 10	Unclear	Primary	Cognitive screening and clinical evaluation—DSM IV and NINCDS-ADRDA
Feldman 2012†	Sweden	Unclear	Population based (HARMONY, KP, and SNAC-K)	Unclear	1021	D, H	ICD 7 ICD 8 ICD 9 ICD 10	304, 305, 306 290, 293.0, 293.1 290.0, 290.1, 290.4, 294.1, 290.8, 290.9, 331.0, 331.1, 331.2, 331.9 F00, F01, F02, F03, G30, G31.1, G31.8, F05.1	Unclear	Dementia diagnoses made in several population-based studies—DSM IV and NINCDS-ADRDA
Henderson 2006	Australia	1998–2001	Population based	Unclear	21	H	ICD 10	F00, F01, F051	Unclear	Auditor coding of hospital records
Preen 2004	Australia	1991–1996	Population based	Unclear	11	H	ICD 9	Unclear	Secondary	Medical record review
Juurlink 2006	Canada	2002–2004	Population based	Unclear	238	H	ICD 10	F03	Unclear	Auditor coding of abstracted medical records
Quan 2008	Canada	2003	Population based	Unclear	Unclear	H	ICD 10	Unclear	Any	Coding from medical record
Nielsen 2011	Denmark	2005–2007	Population based, immigrants only	Median 67	57	H‡	ICD 10	F00.0-9, G30.0-9, F01.0-9, F02.0, F03.9	Any	Medical record review—ICD-10, DSM IV, NINCDS-ADRDA, NINDS-AIREN, McKhann, and McKeith
Phung 2007	Denmark	2003	Population based	Mean 81	197	H‡	ICD 10	F00.0, F00.1, F00.2, F00.9, F01.0, F01.1, F01.2, F01.3, F01.8, F01.9, F02.0, F03.9, G30.0, G30.1, G30.8, G30.9	Any	Clinical evaluation—ICD-10 and DSM IV
Salem 2012	Denmark	2008	Population based, <65 years recruited	∼	195	H‡	ICD 10	F00.0-9, G30.0-9, F01.0-9, F02.0, F03.9, G31.8	Any	Medical record review—ICD-10, DSM IV, NINCDS-ADRDA, NINDS-AIREN, McKhann, and McKeith
Van de Vorst 2015	Netherlands	2006–2010	Population based	Median 80	340	H‡	ICD 9	290.0, 290.1, 290.3, 290.4, 294.1, 331.0, 331.1, 331.82	Any	Medical record review—DSM IV, NINCDS-ADRDA, NINDS-AIREN, McKeith, and McKhann
Dahl 2007	Sweden	Unclear	Population based (GENDER)	Mean 75	35	H	ICD 8 ICD 9 ICD 10	290.0–290.19 290.0–290.9 F00-F03, G30, F10.7, R54	Unclear	Medical record review and cognitive screening—DSM IV
Brown 2016	UK	1997–2008	Population based (MWS)	50–64 at recruitment	244	H	ICD 10	E51.2, F00, F01, F02, F03, F10.6, F10.7, G30, G31.0	Unclear	GP questionnaire
Bender 2016	USA	2009–2012	Heart failure inpatients	Mean 73	44	H	ICD 9	Unclear	Any	Medical record review
Fisher 1992	USA	1984–1985	Population based	Unclear	91	H§	ICD 9	290.0–290.9, 331.0–331.2	Any	Medical record review
Wei 2016	USA	Unclear	Population based	Unclear	100	H‡	ICD 9	Unclear	Unclear	Medical record review
Fujiyoshi 2017	USA	2000–2013	Population based	45–84 at recruitment	306	H & D	ICD 9 ICD 10	290, 294, 331.0, 331.1, 331.2, 331.8, 331.9, 438.0, 780.9 F00, F01, F03, F04, G30, G31.0, G31.1, G31.8, G31.9, I69.9, R41	Any	Medical and research clinic record review
Jaakkimainen 2016	Canada	2010–2011	Population based	>65 at recruitment	Unclear	H, I	ICD 9 ICD 10	46.1, 290.0, 290.1, 290.2, 290.3, 290.4, 294.x, 331.0, 331.1, 331.5, 331.82 F00.x, F01.x, F02.x, F03.x, G30.x	Unclear	Medical record review
Solomon 2014	Finland	1972–2008	Population based (CAIDE)	Mean 79	27	H, M	ICD 8 ICD 9 ICD 10	290, 290.10 290, 291.2, 292.8, 294.1, 331.0, 331.1, 437.8 F00, F01, F02, F03, F05.1, F10.73, F11.73, F14.73, F16.73, F18.73, F19.73, G30	Unclear	Cognitive screening and clinical evaluation—DSM IV and NINCDS-ADRDA
Taylor 2009	USA	1993–2005	Population based (ADAMS)	>70 at recruitment	303	I	ICD 9	331.0, 331.1, 331.2, 331.7, 290.0, 290.1, 290.10, 290.11, 290.1, 209.1, 290.2, 290.2, 290.3, 290.4, 290.4, 290.4, 290.4, 294.0, 294.1, 294.8, 797	Any	Medical records and clinical evaluation—DSM IIIR, DSM IV, NINCDS-ADRDA, NINDS-AIREN, Lund & Manchester, and McKeith
Pippenger 2001	USA	1996–1997	Population based	Unclear	73	O	ICD 9	290.0, 290.1, 290.2, 331.0	Unclear	Medical record review
Dunn 2005	UK	1992–2002	Population based	Mean 82	95	P	Unclear	Unclear	Unclear	GP questionnaire
Heath 2015	UK	Unclear	Population based	40–64 at recruitment	15	P	Read V2	66h.., 6AB.., E00.., E000., E0010, E0011, E0012, E0013, E001z, E002., E0020, E0021, E002z, E003., E004., E0040, E0041, E0042, E0043, E004z, E00y., E00z., E041., Eu00., Eu000, Eu001, Eu002, Eu01., Eu010, Eu011, Eu012, Eu013, Eu01y, Eu01z, Eu02., Eu020, Eu021, Eu022, Eu023, Eu024, Eu025, Eu02y, Eu02z, F110., F1100, F1101, F111., F112., F116., Fyu30	N/A	Medical record review—DSM IV
Butler 2012	USA	2000–2009	Population based	Mean 80	74	P	ICD 9	294.8	Unclear	Medical record review—DSM IV, NINCDS-ADRDA, NINDS-AIREN, and Lund & Manchester

Abbreviations: DNOS, dementia not otherwise specified; D, deaths; H, hospital admissions data; H‡, Hospital admissions and outpatient data set; H§, Hospital admissions data from an insurance data set; M, medications or prescriptions data; O, outpatient data; P, primary care data; I, insurance data; ICD, International Classification of Diseases; PPV, positive predictive value; GP, General Practitioner; CSHA, Canadian Study of Health and Aging; NEDICES, Neurological Diseases in Central Spain; HARMONY, Study of Dementia in Swedish Twins; KP, Kungsholmen Project; SNAC-K, Swedish National Aging and Care Study in Kungsholmen/Essingeöarna; CAIDE, Cardiovascular Risk Factors, Aging and Dementia; GENDER, A Study of Older Unlike-Sex Twins; ADAMS, Aging Demographics and Memory Study; MWS, Million Women Study; DSM, Diagnostic and Statistical Manual; NINCDS-ADRDA, National Institute of Neurological and Communicative Disorders and Stroke and the Alzheimer's Disease and Related Disorders Association, NINDS-AIREN, National Institute of Neurological Disorders and Stroke Association and Association Internationale pour la Receherché et l'Enseignement en Neurosciences; McKeith, McKeith et al. consensus guidelines for dementia with Lewy bodies (1996); McKhann, McKhann et al. report of the Work Group on Frontotemporal Dementia and Pick's Disease (2001); Lund & Manchester, criteria for frontotemporal dementia from Lund and Manchester groups (1994).

NOTE. Some study used clinically modified versions of ICD coding system which extends code length to provide extra detail (i.e., ICD-9-CM); however, for the purposes of dementia coding up to four digits, these are identical to the original versions.

NOTE. Ampersand (&) between data sets indicates >1 data sets were combined for the analysis, and commas (,) indicate data sets were analyzed separately, producing separate PPV figures. Drug codes were not provided in either study that assessed medication data sets.

NOTE. Studies ordered by routine data set type.

∗ Any information given regarding the ages of dementia cases or age at recruitment. Study period: years from which coded data were obtained. Study size corresponds to the number of coded dementia cases (true positives and false positives).

† Abstract from conference poster presentation only, full study not yet published.

**Table 2:** Studies reporting the sensitivity of routinely collected data sets for the ascertainment of dementia cases

First author and publication year	Country	Study period	Study population	Age∗	Method of dementia case identification or confirmation and diagnostic criteria if used	Study size	Routine data set	Coding system	Code(s) assessed	Coding position
Ostbye 1999 [6]	Canada	1991–1996	Participants from CSHA, a randomly selected group of elderly people across Canada	>65 at recruitment	Screening followed by neurologic and neuropsychological examinations—DSM III and NINCDS-ADRDA	452	D	ICD 9	331.0, 290.0–290.3, 290.8–290.9, 290.4, 291.2, 291.8, 294.0–294.9, 332.0–332.1, 333.4. 797	Any
Romero 2014 [8]	Spain	1993–2007	NEDICES survey—a longitudinal population-based survey of people aged >65 years within three communities	Mean 82	Cognitive screening followed by clinical evaluation—DSM IV and NINCDS-ADRDA	403	D	ICD 9 ICD 10	Unclear	Primary
Feldman 2012†[9]	Sweden	Unclear	Population-based twin study (HARMONY)	Unclear	Participant screening via telephone or in-person testing followed by clinical work-ups—DSM IV and NINCDS-ADRDA	559 526 526	H D H & D	ICD7 ICD8 ICD9 ICD10	304, 305, 306 290, 293.0, 293.1 290.0, 290.1, 290.4, 294.1, 290.8, 290.9, 331.0, 331.1, 331.2, 331.9 F00, F01, F02, F03, G30, G31.1, G31.8, F05.1	Unclear
Jin 2004 [27]	Sweden	1987–2000	Participants in SATSA and OCTO-Twin studies	Mean 81	Baseline assessment then telephone screening and clinical evaluation during follow-up—DSM IV, NINCDS-ADRDA, and NINDS-AIREN	321 269 321	H D H & D	ICD 8 ICD 9 ICD 10	290, 290.10, 290.11, 290.19, 293 290A, 290B, 290E, 290W, 290X, 294B, 331A A81.0, F00.0, F00.1, F00.2, F00.9, F01.0, F01.1, F01.2, F01.3, F01.8, F01.9, F02.0, F02.1, F02.3, F02.8, F03.9, F05.1, G30.0, G30.1, G30.8, G30.9, G31.0, G318 A	Any
Newens 1993 [26]	UK	1986–1992	Early-onset dementia cases identified through hospital records and via inquires to social services, day hospitals, psychiatric nurses, nursing homes, psychologists, general practitioners, and neuroradiology centers	40–64 at recruitment	Medical record review and clinical algorithm	257	D	ICD 9	Unclear	Any
Solomon 2014 [14]	Finland	1972–2008	CAIDE study-derived from 4 population-based random samples	Mean 79	Cognitive screening followed by clinical evaluation and then case conference—DSM IV and NINCDS-ADRDA	51 52	H M	ICD 8 ICD 9 ICD 10	290, 290.10 290, 2912A, 2928C, 2941A, 3310A, 3311A, 4378A F00, F01, F02, F03, F05.1, F10.73, F11.73, F14.73, F16.73, F18.73, F19.73, G30	Unclear
Dahl 2007 [16]	Sweden	Unclear	Unlike-sex twins born between 1916–1925 and both twins alive at 1995 identified through Swedish Twin Registry	Mean 75	Cognitive screening and medical record review and then case conference—DSM IV	87	H	ICD 8 ICD 9 ICD 10	290.0–290.19 290.A–290.X F00-F03, G30, F10.7, R54	Unclear
Taylor 2009 [22]	USA	1993–2005	ADAMS study—a stratified random sample of respondents to the Health and Retirement Study	>70 at recruitment	Medical record review, informant history, and clinical evaluation—DSM IIIR, DSM IV, NINCDS-ADRDA, NINDS-AIREN, Lund & Manchester, and McKeith	275	I	ICD 9	331.0, 331.1, 331.2, 331.7, 290.0, 290.1, 290.10, 290.11, 290.12, 209.13, 290.20, 290.21. 290.3, 290.40, 290.41, 290.42, 290.43, 294.0, 294.1, 294.8, 797	Any

Abbreviations: H, hospital admissions; D, deaths; M, medications or prescriptions; I, insurance; H & D, hospital and death data combined; ICD, International Classification of Diseases; CSHA, Canadian Study of Health & Ageing; NEDICES, Neurological Diseases in Central Spain; HARMONY, Study of Dementia in Swedish Twins; SATSA, Swedish Twin Registry who took part in the Swedish Adoption/Twin Study of Ageing; OCTO-Twin, Origins of Variance in the Oldest Old; CAIDE, Cardiovascular Risk Factors, Aging and Dementia; ADAMS, Aging Demographics and Memory Study; DSM, Diagnostic and Statistical Manual; NINCDS-ADRDA, National Institute of Neurological and Communicative Disorders and Stroke and the Alzheimer's Disease and Related Disorders Association; NINDS-AIREN, National Institute of Neurological Disorders and Stroke Association and Association Internationale pour la Receherché et l'Enseignement en Neurosciences; McKeith, McKeith et al. consensus guidelines for dementia with Lewy bodies (1996); McKhann, McKhann et al. report of the Work Group on Frontotemporal Dementia and Pick's Disease (2001); Lund & Manchester, criteria for frontotemporal dementia from Lund and Manchester groups (1994).

NOTE. Drug codes not available for study that assessed medications data set.

NOTE. Some studies used clinically modified versions of ICD coding system which extends code length to provide extra detail (i.e., ICD-9-CM); however, for the purposes of dementia coding up to four digits, these are identical to the original versions.

NOTE. Study period: years from which coded data were obtained. Study size: total number of patients known to have dementia (true positives and false positives combined).

NOTE. Studies ordered by routine data set type.

∗ Any information given regarding the ages of dementia cases or age at recruitment. Studies either attempted to ascertain all dementia cases within a cohort of participants, or attempted to ascertain all dementia cases within a geographical population and then verified these diagnoses.

† Abstract from conference poster presentation only, full study not yet published.

All studies were performed in high-income countries: 10 in mainland European countries [7], [8], [9], [14], [15], [16], [17], [18], [25], [31], four in the UK [19], [28], [29], [32], 11 in North America [6], [12], [13], [20], [21], [22], [23], [24], [26], [27], [30], and two in Australia [10], [11]. Studies varied widely with respect to population characteristics, data set type, coding system and version, codes used to select cases, and the reference standard to which coded data were compared. Most studies identified cases from a defined general population, but one involved nursing home residents [7], and another was of hospitalized patients with heart failure [20]. Only 12 of the 27 studies reported the average age of dementia cases (range 58–85 years), while a further seven only stated the ages of participants at recruitment. Most studies investigated hospital data (variably including hospital admissions with or without outpatient appointments) or death data [6], [7], [8], [9], [10], [11], [12], [13], [14], [15], [16], [17], [18], [19], [20], [21], [22], [23], [24], [25], [27], [31], [32]. Two studies assessed insurance data [24], [26], three assessed primary care data [28], [29], [30], and one assessed prescription data [25].

Studies investigated all-cause dementia [6], [7], [8], [9], [10], [11], [13], [14], [15], [16], [17], [18], [19], [20], [21], [22], [23], [24], [25], [26], [27], [28], [29], [30], [31], [32], AD [6], [9], [15], [16], [23], [25], [26], [31], vascular dementia [6], [9], [15], [16], [17], [23], [31], or unspecified dementia only [12]. No studies investigated other dementia subtypes, such as frontotemporal dementia or dementia with Lewy Bodies. Studies varied in the codes selected to identify dementia and subtype cases (Supplementary Appendix C).

The reference standards to which coded data were compared varied. They could be broadly categorized as follows: direct clinical evaluation [6], [7], [9], [15], [26], cognitive screening followed by clinical evaluation [8], [25], [31], medical record review [10], [11], [12], [13], [14], [16], [17], [18], [20], [21], [22], [23], [24], [27], [29], [30], [32], or a General Practitioner questionnaire [19], [28].

### 3.2 Quality assessment

Only five studies [15], [17], [18], [23], [24] were judged as having a low risk of bias and applicability concerns across all categories (Supplementary Appendix D). Most studies had one or more “unclear” ratings across categories, either because information was not provided or was unclear in the publication. Eight studies that assessed PPV had a high risk of bias or applicability concerns in one or more areas [7], [14], [16], [19], [20], [22], [29], [30], but no studies of sensitivity were so affected.

### 3.3 PPV–all-cause dementia

**Fig. 2:**
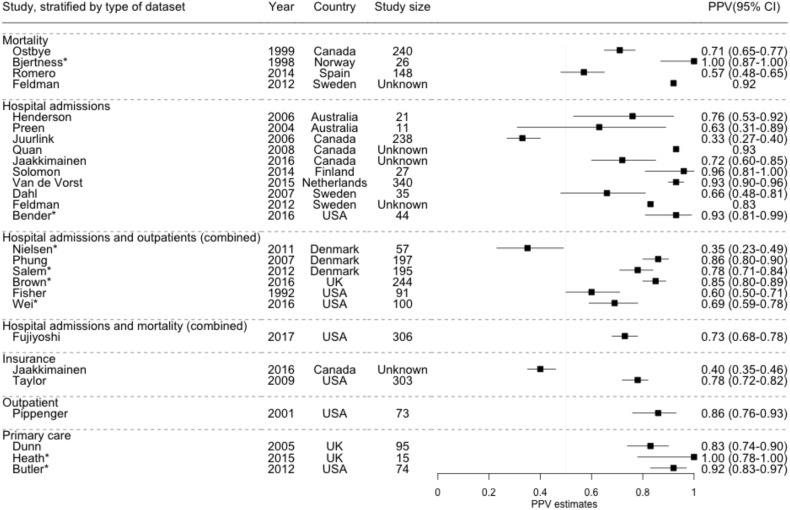
PPV estimates for routinely collected coded health data to identify all-cause dementia cases, stratified by type of routine data set. Study size: number of cases with ≥1 dementia codes in data set. *High risk of bias or applicability concerns in one or more areas. Abbreviations: PPV, positive predictive value; CI, confidence interval.

For all-cause dementia, there were 27 PPV estimates in total (Fig. 2). Four studies reported the PPV for dementia coding in mortality data [6], [7], [8], [9], 10 in hospital admissions data bib10[9–13,17,18,20,24,25], six in hospital admissions and outpatient data combined [14], [15], [16], [17], [19], [21], [22], one in hospital admissions and mortality combined [23], one in outpatient data [27], two in insurance data [24], [26], and three in primary care data [28], [29], [30]. Although results varied widely, with PPVs ranging from 33%–100% across all studies, 16 of the 27 PPV estimates were >75%. Of the eight studies at high risk of bias, four reported very high PPVs [7], [20], [29], [30], and one reported a low PPV of 35% [14], raising the possibility that results at the extremes of the range of reported estimates may be partly due to bias. Visual inspection of the forest plot revealed no clear differences between data set types. The three primary care studies reported PPVs of 83%, 92%, and 100% [28], [29], [30]. There was no clear difference between studies when stratified by the method of reference standard used (Supplementary Appendix E).

### 3.4 PPV–Alzheimer's disease and vascular dementia

**Fig. 3:**
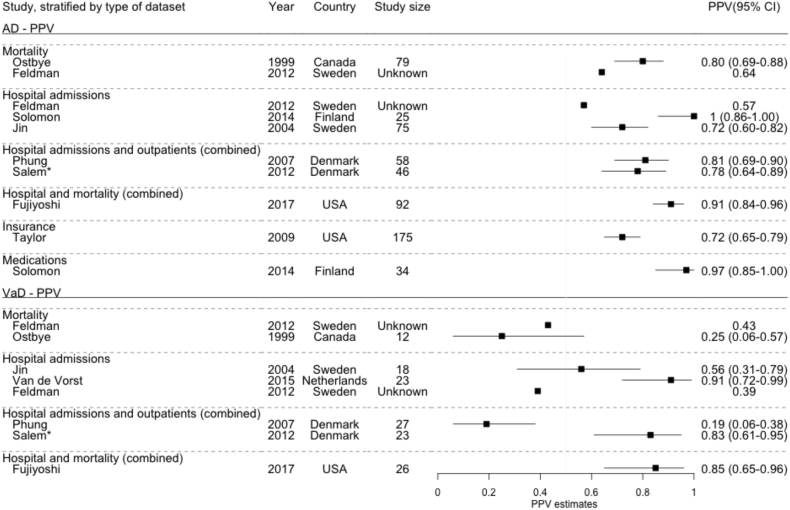
PPV estimates for routinely collected coded health data to identify dementia subtype cases, stratified by type of routine data set. Study size: number of cases with ≥1 dementia codes in data set. *High risk of bias or applicability concerns in one or more areas. Abbreviations: AD, Alzheimer's disease; VaD, vascular dementia; PPV, positive predictive value; CI, confidence interval.

There were 10 estimates of PPV for coding for AD [6], [9], [15], [16], [23], [25], [26], [31] and eight for vascular dementia [6], [9], [15], [16], [17], [23], [31] (Fig. 3). PPVs for AD (range 57%–100%) were generally higher and less variable than those for vascular dementia (range 19%–91%). Six studies provided estimates of PPV for AD and vascular dementia [6], [9], [15], [16], [23], [31]. Five of these found a higher PPV for coding of AD compared with vascular dementia. A single study of the accuracy of using codes for prescriptions of AD medications to identify AD cases reported a high PPV (97%) [25].

### 3.5 Sensitivity–all-cause dementia

**Fig. 4:**
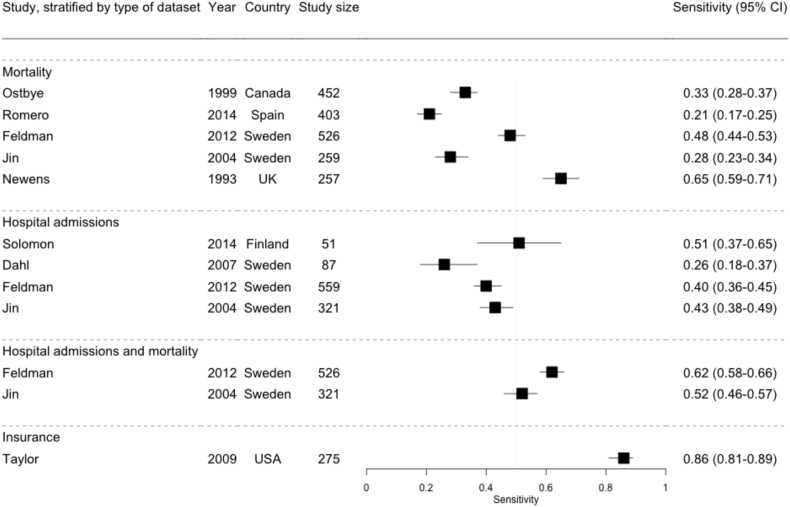
Sensitivity estimates for routinely collected coded health data to identify all-cause dementia cases, stratified by type of routine data set. Study size: Number of known dementia cases for which a code was sought. Abbreviation: CI, confidence interval.

The 12 estimates of sensitivity for all-cause dementia ranged from 21%–86%, with only three studies reporting estimates >60% (Fig. 4). The only study investigating insurance data reported the highest sensitivity (86%), likely reflecting the comprehensive coverage of this data source [26]. The lowest sensitivity (21%) came from a study which only selected codes in the primary position on the death certificate [8]. There were no clear overall differences in sensitivity of hospital and mortality data, but two studies demonstrated higher sensitivities from combining hospital admissions and mortality data compared with either source alone, increasing from 48% and 28% in mortality data and from 40% and 43% in hospital admissions data to 62% and 52% in both sources combined [9], [31].

### 3.6 Within-study analyses

Supplementary Appendix F shows results of 10 within-study analyses from seven studies [6], [8], [15], [17], [19], [21], [32]. In general, sample sizes were small, resulting in broad confidence intervals. Selecting codes only in the primary versus any other position gave a higher PPV but, unsurprisingly, at a cost to sensitivity, with fewer cases identified [6], [21], [32]. The results of two studies suggested that relying on codes that refer to dementia subtypes (such as AD and/or vascular dementia) to identify any dementia case (not necessarily that subtype) produced a higher PPV than using general dementia codes but with fewer cases identified [6], [15]. In keeping with the positive association between PPV and disease prevalence, one study demonstrated a lower PPV for patients <65 versus ≥65 years of age (PPV 68% and 96%, respectively) [17]. One study reported that death certificates identified moderate or severe dementia with a higher sensitivity than mild dementia [8]. Finally, one study found that patients with ≥2 dementia codes in hospital admissions data were more likely to have dementia than those with only one code (PPV 94% vs. 68%, respectively) [19].

## 4 Discussion

### 4.1 Summary of findings

In this systematic review, we found wide variation in the results of validation studies of dementia coding in routinely collected health-care data sets, at least partly reflecting the heterogeneity in study methodologies, settings, and the data sets they assessed. Importantly, however, we found that in some settings, these data sets can achieve high PPVs of >80%–90%. By contrast, the sensitivity of the data sets investigated to date is lower, with many data sources identifying <50% of all dementia cases.

For all-cause dementia, primary care data appears to identify cases with a high PPV [28], [29], [30]. Combining hospital and death data produces a reasonable sensitivity for all-cause dementia [9], [31], and, of the data sources assessed, the US insurance data produces the highest sensitivity [26]. For identifying AD cases, PPV is reassuringly high across most studies and appears to be particularly high in medications data [25] and combined US hospital and mortality data [23].

There is no widely accepted minimum level of accuracy for disease case ascertainment in prospective studies. The level of accuracy that is considered acceptable is likely to differ according to the study setting, and there will inevitably be a trade-off between PPV and sensitivity. For example, large prospective studies are likely to be best served by data sources which achieve a high PPV even if these data sets have a lower sensitivity, as the number of false positives (controls misidentified as dementia cases) must be minimized to reduce bias and distortion of risk estimates [33]. A high sensitivity is less crucial because the effects of false negatives (cases misidentified as controls) would be diluted among the large control population. A reasonable sensitivity is still required, however, to ensure that the cases ascertained are representative and to maximize statistical power.

Variation in study methodologies may explain some of the wide variation in PPV estimates. For example, the two studies with the lowest PPV estimates investigated only a single code (F03—unspecified dementia) and an ethnic minority population, respectively [12], [14]. In one of these, the PPV was likely to have been further lowered by high rates of “indeterminate” cases due to the particularly strict reference standard requirements [14]. By contrast, one study with the joint-highest PPV involved nursing home residents only, a population with a high prevalence of dementia, which will increase PPVs because of the positive association between PPV and disease prevalence [7]. Furthermore, the reference standards used varied widely with respect to which, if any, diagnostic criteria were employed and whether the diagnosis was made by screening followed by in-person evaluation, medical record review, General Practitioner questionnaire, or another method.

The sensitivity of routine data sets for identifying dementia cases appeared lower than that of some other neurodegenerative diseases, such as motor neurone disease [34]. Key differences between these conditions may explain the lower sensitivity of dementia coding. First, it is recognized that a significant proportion of dementia cases are undiagnosed and so missing from routine data sets [35], [36]. This is less of an issue for conditions such as motor neurone disease that result in rapidly progressive physical symptoms. Second, for patients with a diagnosis of dementia, their dementia may not be the primary reason for admission to hospital, meaning it may not be mentioned in hospital discharge summaries and so omitted from hospital admissions data [37]. However, the sensitivity of routinely collected health-care data is changing over time. For example, a UK clinic-based study reported an improvement in the sensitivity of mortality data for dementia from 40% to 63% between 2006 and 2013, probably reflecting a changing awareness and desire to diagnose dementia in health professionals, patients, and caregivers over time [38].

### 4.2 Future directions—improving accuracy of dementia identification

Given that management of dementia is predominantly community based, primary care data sets may provide an opportunity to identify cases that do not appear in hospital admissions or mortality data. Three small studies reported on the PPV of primary care data sets, and these suggested that primary care data may identify dementia cases with a high PPV, in keeping with our previous findings that primary care can be an accurate data source for other neurodegenerative diseases [34]. This warrants further investigation. Our review also identified a need for studies of the accuracy of routinely collected health-care data to identify dementia subtypes other than AD or vascular dementia (e.g., frontotemporal dementia or dementia with Lewy bodies).

The use of medication prescription data to identify AD cases is an under-investigated area, but one small study reported a promising PPV of 97% [25]. Dementia drugs such as cholinesterase inhibitors are now commonly prescribed for patients with dementia with Lewy bodies and for AD and therefore medication data alone may not be sufficiently accurate to identify dementia subtypes. Although the indications for these medications are relatively specific to dementia, they may be used in other conditions, such as memantine for migraine [39]. Future studies with larger sample sizes would allow further evaluation of medication data to identify AD and all-cause dementia.

Cohorts may wish to link to several different data sets to increase sensitivity. To date, only hospital admissions and death registrations have been evaluated in combination. Studies investigating the accuracy of using combinations of data sets (e.g., primary care, hospital admissions, and death data together) are required to pursue this further.

Case detection algorithms need to achieve an appropriate balance between the proportion of cases that are true positives (high PPV) and comprehensive case ascertainment (high sensitivity). Results from the within-study analyses reported here provide some possible mechanisms through which cases can be identified with a high PPV. For example, we found higher PPVs when all-cause dementia cases were identified using codes for dementia subtypes compared with general dementia codes [6], [15], [19], by selecting dementia codes in the primary position rather than other positions [6], [21], or by requiring a dementia diagnosis code to occur in ≥2 rather than only one hospital admission [19]. However, in each of these studies, the use of these techniques to increase PPV reduced the number of cases identified.

One method of maximizing both PPV and sensitivity may be to use a broad code list to identify cases from routinely collected data, followed by an examination of the full-text medical records to select participants who truly have dementia. Whereas, this would be time consuming to do manually in a large study, the use of natural language processing to confirm diagnoses of dementia from free-text records holds promise [40]. One study found that combining natural language processing with coded data produced a PPV of 92% [41].

### 4.3 Strengths and limitations

We used rigorous systematic review methodology to maximize the validity of our results. This included prospective protocol publication; detailed search criteria; and duplication of study screening, quality assessments, and data extraction by two authors.

There were some limitations however. First, Quality Assessment of Diagnostic Accuracy Studies 2 assessment showed that studies were of variable quality with some risk of bias. Second, publication bias (with a possible tendency to publish results demonstrating good accuracy) may also have influenced our results. We did not attempt to quantify this due to the absence of a robust technique for doing so in test accuracy reviews [42]. Third, PPV increases with disease prevalence and so studies in settings with a higher prevalence of dementia (older populations and care home residents) will inevitably result in higher PPVs. We could not formally adjust for the underlying prevalence of dementia in the study populations, but rather attempted to take this into account in interpreting the results. Fourth, we included all relevant studies published since 1990, but results from the older studies among these may be of less contemporary relevance because perceptions and diagnostic boundaries of dementia have changed over time. Fifth, many studies reported a relatively young average age of dementia cases (e.g., <80 years), limiting the generalizability of the findings to studies ascertaining dementia in the oldest old.

A major source of heterogeneity in validation studies, and therefore a limitation of our systematic review, is the variation in the reference standards to which the coded data were compared. This reflects the complexities of dementia diagnosis and the lack of a robust “gold standard” for confirmation of cases in dementia research [43]. Although we did not see a pattern in reported PPVs when stratifying by reference standard, it is highly likely that the method of case confirmation will affect study estimates. Similarly, studies differed on whether diagnostic criteria were applied during validation, and the use of strict diagnostic criteria is likely to affect the study estimates. Future studies will need to carefully consider the reference standard used and could consider reporting a “best case” and “worst case” PPV, based on how strictly diagnostic criteria are applied.

## 5 Conclusion

Although no replacement for in-person, comprehensive clinical assessment, routinely collected health-care data sets have the potential to be a cost-effective and comprehensive method of identifying dementia cases in prospective studies. Given the marked heterogeneity between existing validation studies, cohorts should ideally validate these data sets using their own data so that the accuracy is known for their specific study population and setting. Dementia subtypes, primary care, prescribing data, and the development of algorithms to maximize accuracy are potentially useful and under-investigated areas for further research.

**Research in Context**

1. Systematic review: We searched the databases MEDLINE, EMBASE, Web of Science, CENTRAL, and PsycINFO for studies in which the coding of dementia cases in routinely collected data sets was compared with an expert-led reference standard and either positive predictive value (PPV) or sensitivity estimates were reported or calculable.
2. Interpretation: We found a wide range of methodologies used by validation studies. Most studies validated hospital and/or death data, three investigated primary care data, two evaluated insurance data, and single studies assessed prescription and outpatient data. Reported estimates for PPV and sensitivity varied widely, but many studies achieved high PPV and/or reasonable sensitivity. Coding for Alzheimer's disease had generally higher and more consistent PPVs than for vascular dementia.
3. Future directions: Identification of dementia subtypes, the accuracy of primary care and prescription data, and the development of algorithms to maximize accuracy are promising but under-investigated areas for future research.
