# Supplementary material for: Identifying dementia cases with routinely collected health data: A systematic review
Source: Alzheimers Dement. 2018 Aug;14(8):1038–51. doi: 10.1016/j.jalz.2018.02.016 (PMC6105076; doi:10.1016/j.jalz.2018.02.016)
Supplement: Appendix A [file mmc1.docx]

**Appendix A. Search strategy**

**MEDLINE search strategy (Ovid MEDLINE(R) In-Process & Other Non-Indexed Citations and Ovid MEDLINE(R) 1946 to Present)**

| 1 | "International Classification of Diseases"/ or "international classification of diseas*".mp. or "ICD ten*".mp. or ICD10.mp. or "ICD 10".mp. or "ICD 9".mp. or ICD9.mp. or "ICD nine".mp. or ICD-9-CM.mp. or ICD-10-CM.mp. or "administrat* data".mp. or "medical record*".mp. or "health information*".mp. or claim*.mp. or "hospital discharge*".mp. or "inpatient discharge*".mp. or "hospital episode*".mp. or "hospital episode statistics".mp. or "scottish morbidity record*".mp. or SMR*.mp. or "patient episode database for wales".mp. or PEDW.mp. or coding.mp. or code*.mp. or exp Clinical Coding/ or "medical record review".mp. or exp Information Systems/ or exp Medical Records/ or exp medical records systems, computerized/ or exp electronic health records/ or exp Electronic Health Records/ or exp Primary Health Care/ or exp general practice/ or exp family practice/ or "read cod*".mp. or exp Patient Discharge/ or exp Patient Discharge Summaries/ or exp Hospital Records/ or exp Health Services Research/ or "physician claims".mp. or "death certificate*".mp. or exp death certificates/ or exp hospital records/ or "death registration*".mp. or medicare.mp. or exp health insurance/ or exp Outpatients/ or "diagnostic and statistical manual".mp. or dsm*.mp. or "case ascertainment".mp. |
| --- | --- |
| 2 | (sensitivity or specificity).mp. or exp "sensitivity and specificity"/ or ((pre-test or pretest) adj probability).mp. or exp "Predictive Value of Tests"/ or "predictive value*".mp. or "likelihood ratio*".mp. or exp validation studies/ or "validation stud*".mp. or "positive predictive value".mp. or exp "reproducibility of results"/ or "reproducibility of results".mp. or "positive predictive value".mp. or "negative predictive value".mp. or validity.mp. or reproducibility.mp. or accuracy.mp. or agreement.mp. or validation.mp. or algorithm*.mp. or exp algorithms/ or (identif* adj3 dement*).ti,ab. or (detect* adj3 dement*).ti,ab. or (ROC or "receiver operat*").ab. or sROC.ab. or Area Under Curve/ |
| 3 | dementia.mp. or exp Dementia/ or exp Alzheimer Disease/ or alzheimer*.mp. or "vascular dementia".mp. or exp Dementia, Vascular/ or exp Dementia, Multi-Infarct/ or "lewy body".mp. or exp Lewy Bodies/ or exp Frontotemporal Lobar Degeneration/ or exp Frontotemporal Dementia/ or frontotemporal.mp. or exp Cognition Disorders/ or neurocognitive.mp. or exp Cognition/ or exp Delirium, Dementia, Amnestic, Cognitive Disorders/ |
| 4 | 1 and 2 and 3 |
| 5 | limit 4 to yr="1990-Current” |

**EMBASE search strategy – (Embase 1980 to Present)**

| 1 | "International Classification of Diseases"/ or disease classification/ or icd-10/ or icd-10-cm/ or icd-10-pcs/ or icd-9/ or icd-9-cm/ or "administrat* data".mp. or "medical record*".mp. or medical information system/ or medical informatics/ or electronic medical record/ or "health information".mp. or medical record/ or "hospital discharge*".mp. or claim*.mp. or "inpatient discharge*".mp. or "hospital episode*".mp. or "hospital episode statistics".mp. or "scottish morbidity record*".mp. or SMR.mp. or SMR01.mp. or "patient episode database for wales".mp. or PEDW.mp. or coding algorithm/ or patient coding/ or coding/ or coding.mp. or code*.mp. or "medical record review".mp. or exp medical record/ or exp electronic medical record/ or exp primary health care/ or "read cod*".mp. or "hospital record*".mp. or exp death certificate/ or registration/ or medicare.mp. or exp health insurance/ or exp Outpatients/ or "outpatient data".mp. or dsm*.mp. |
| --- | --- |
| 2 | "sensitivity and specificity"/ or exp predictive value/ or exp accuracy/ or validity/ or predictive validity/ or validation.mp. |
| 3 | exp dementia/ or alzheimer disease/ or diffuse lewy body disease/ or frontotemporal dementia/ or multiinfarct dementia/ or presenile dementia/ or senile dementia/ or Lewy body/ or exp frontotemporal dementia/ |
| 4 | 1 and 2 and 3 |
| 5 | limit 4 to yr="1990-Current" |

**Web of Science search strategy – (Web of Science Core Collection)**

| 1 | TS=(“international classification of diseases” OR “ICD*” OR “administrat* data” OR “medical record*” OR “health information” OR claim* OR “hospital discharge*” OR “inpatient discharge*” OR “hospital episode*” OR “hospital episode statistics” OR “Scottish morbidity record” OR “patient episode database for wales” OR coding OR code* OR “medical record*” OR “electronic health record*” OR “hospital record*” OR medicare OR “health insurance”) |
| --- | --- |
| 2 | TS=(“positive predictive value” OR “negative predictive value” OR accuracy OR sensitivity OR specificity OR validity) |
| 3 | TS=dementia |
| 4 | 1 and 2 and 3 (*Timespan=1990-2017)* |

**PsycINFO search strategy – PsycINFO 1987 to current**

| 1 | exp International Classification of Diseases/ or administrat* data.mp. or exp Medical Records/ or exp Hospitalized Patients/ or exp Primary Health Care/ or exp Hospital Discharge/ or exp Databases/ or coding.mp. or code*.mp. or administrat* data.mp. or scottish morbidity record*.mp. or SMR*.mp. or hospital episode statistics.mp. or HES.mp. or electronic health record.mp. or medicare or exp health insurance/ or exp Outpatients/ or outpatient data.mp. |
| --- | --- |
| 2 | exp Test Validity/ or exp Test Reliability/ or exp Diagnosis/ or positive predictive value.mp. or negative predictive value.mp. or sensitivity.mp. or specificity.mp. or exp Statistical Validity/ or validity.mp. or accuracy.mp. |
| 3 | exp Vascular Dementia/ or exp Dementia/ or exp Semantic Dementia/ or exp Presenile Dementia/ or dementia.mp. or exp Dementia with Lewy Bodies/ or exp Senile Dementia/ |
| 4 | 1 and 2 and 3 |
| 5 | limit 4 to yr="1990-Current" |

**Cochrane Library search strategy – (Cochrane Reviews (Reviews only), Other Reviews, Trials, Methods Studies, Technology Assessments, Economic Evaluations and Cochrane Groups)**

| 1 | “international classification of diseases” OR “ICD*” OR “administrat* data” OR “medical record*” OR “health information” OR claim* OR “hospital discharge*” OR “inpatient discharge*” OR “hospital episode*” OR “hospital episode statistics” OR “Scottish morbidity record” OR “patient episode database for wales” OR coding OR code* OR “medical record*” OR “electronic health record*” OR “hospital record*” OR medicare OR “health insurance” |
| --- | --- |
| 2 | “positive predictive value” OR “negative predictive value” OR accuracy OR sensitivity OR specificity OR validity |
| 3 | dementia OR alzheimer OR “vascular dementia” |
| 4 | 1 and 2 and 3 (Year from 1990 to 2017) |
