# Supplementary material for: Identifying dementia cases with routinely collected health data: A systematic review
Source: Alzheimers Dement. 2018 Aug;14(8):1038–51. doi: 10.1016/j.jalz.2018.02.016 (PMC6105076; doi:10.1016/j.jalz.2018.02.016)
Supplement: Appendix B [file mmc2.docx]

**Appendix B. Adapted QUADAS-2 assessment form**

Primary study:

Flowchart:

1. **Patient selection**

| 1. Risk of bias: *Could the selection of patients have introduced bias?* | |
| --- | --- |
| Describe methods of patient selection: *Consider whether the criteria used to identify dementia cases will pick up a consecutive or random sample or all cases within a specific subpopulation e.g. a certain geographical region. To what extent does this subpopulation reflect the wider population (study more generalisable if has a wider area of coverage)*  *Does the study only include patients presenting in a certain manner, or with a specific reason for contact with the health system (e.g. nursing home etc.) or patients with a given severity of disease. What age of patients are included? Does the method of patient selection aim to pick up incident or prevalent cases? Is the population at risk well defined? (accurate demographic information about the population at risk must be available so that an appropriate denominator can be used) Who identified patients and who ensured that selection adhered to patient recruitment criteria?* | |
| Was a consecutive or random sample of patients enrolled? | Yes / No / Unclear |
| Was a case-control design avoided? | Yes / No / Unclear |
| Did the study avoid inappropriate exclusions?  *Does the study avoid excluding patients whose principal diagnosis/ presenting complaint was not related to dementia, but who had dementia as a co-morbidity?* | Yes / No / Unclear |
| Could the selection of patients have introduced bias?  Risk: Low/ High/ Unclear | |

| 1. Applicability assessment: *Are there concerns that the included patients and setting do not match the review question?* |
| --- |
| Describe methods of patient selection and included patients. *Consider if patients with other conditions (e.g. delirium, mild cognitive impairment or other non-dementia cognitive disorders) or if healthy individuals may be identified and/or included in the group of cases. Does the setting of the study and the demographic features of the study cohort reflect the wider population? Is the study population large enough? Does the method of patient selection aim to pick up incident or prevalent cases?* |
| Is there concern that the included patients do not match the review question?  Concern: Low / High / Unclear |

1. **Index test**

| 1. Risk of bias: *Could the conduct or interpretation of the index test have introduced bias?* | |
| --- | --- |
| Describe the index test and how it was conducted and interpreted: *Consider if the index test may introduce bias e.g. are presentations to primary care likely to be less severe/complex than hospital presentations and what effect will this have on making the diagnosis? For what reason(s) have the individuals been recorded in the administrative dataset? Does the study avoid only including presentations to healthcare that are likely to occur in earlier or later stages of the disease? Does the study only include first hospital admissions/presentations, or does it also include repeat admissions/presentations? If hospital data investigated describe the features of the hospital, as they may have some influence on the diagnostic accuracy; size of hospital, location, teaching hospital, presence of neuro dept.; public/private hospital clinical training of the person making the diagnosis or performing the diagnostic coding etc. Were only certain wards included and does hospital data include only discharge data, or only outpatient data, or possibly both? Similar variables to those outlined above will need to be considered and described for data from general practice and possibly also from death registrations. Is mortality data based on ‘underlying’ and ‘contributory’ cause?* | |
| Were the index test results interpreted without knowledge of the results of the reference standard? | Yes / No / Unclear |
| If a threshold was used, was it pre-specified?  *Consider if the study includes possible/probable cases of dementia +/- subtypes* | Yes / No / Unclear |
| Did the study avoid inappropriate exclusions?  *See above. Consider what influences presentation of patients with dementia to GP/ hospital and mortality data* | Yes / No / Unclear |
| Could the conduct or interpretation of the index test have introduced bias?  Risk: Low / High / Unclear | |

| 1. Applicability assessment: *Are there concerns that the index test, its conduct, or interpretation differ from the review question?* |
| --- |
| *See above. Does the index test conform to the definition of a routinely collected dataset?*  Concern: Low / High / Unclear |

1. **Reference standard**

| 1. Risk of bias: *Could the reference standard, its conduct, or its interpretation have introduced bias?* | |
| --- | --- |
| Describe the reference standard and how it was conducted and interpreted: *The reference standard could be a retrospective medical note review, autopsy or known dementia disease registry amongst others.* *Is the gold standard classification performed by somebody with sufficient knowledge and experience to make this diagnosis accurately? Similarly, a sufficient amount of high quality medical documentation is necessary for retrospective review of clinical documentation. Did the study describe the characteristics of the assessor(s) examining the reference standard? Was the clinician who performed the assessment blinded to the results of the index test? If an ‘expert clinician’ performed the evaluation, what level of training and expertise do they have? Did the study involve more than one assessor and if so do they have the same level of expertise?*  *If >1 person reading reference standard what was the level of agreement?* | |
| Is the reference standard likely to correctly classify the target condition? | Yes / No / Unclear |
| Were the reference standard results interpreted without knowledge of the results of the index test? | Yes / No / Unclear |
| Could the reference standard, its conduct, or its interpretation have introduced bias?  Risk: Low / High / Unclear | |

| 1. Applicability assessment: Are there concerns that the target condition as defined by the reference standard does not match the question? |
| --- |
| Describe the reference standard and how it was conducted and interpreted: *See above. Is the gold standard used to classify the degree of diagnostic certainty into probable/possible/definite dementia appropriate? Which, if any, diagnostic criteria were used and are these appropriate? Which groups are included in statistical analysis? For example including cases of uncertain diagnosis may significantly affect PPV and sensitivity. If a disease register is used, is it well established and has it been shown to have ~100% case ascertainment; for the first few years following establishment case identification may still be low.*  Concern: Low / High / Unclear |

1. **Flow and timing**

| 1. Risk of bias: *Could the patient flow have introduced bias?* | |
| --- | --- |
| Describe any patients who did not receive the index test(s) and/or reference standard or who were excluded from the 2x2 table (refer to flow diagram):  *Did the study describe number of pts who satisfied inclusion/exclusion criteria? Is a study flow diagram presented? Can all patients be followed through the study? Are any patients not included in analysis that were originally identified? If so, why? For example exclusion of patients based on residency may not be appropriate. If data from general practice or hospital discharges used, excluding patients who die before the end of the study may not be appropriate. Consider what ages included/excluded; in studies based on mortality data. On the other hand it reduces the likelihood of false-positive cases.* *The exclusion of patients whose medical notes cannot be retrieved may introduce bias.* | |
| Describe the time interval and any interventions between index test(s) and reference standard: | |
| Was there an appropriate interval between index test and reference standard?  *For example are the reference cases obtained from the same time period as that over which the health datasets are assessed? Using populations from different time periods and assuming that the incidence and prevalence are the same would be a flaw.* | Yes / No / Unclear |
| Did all patients receive a reference standard? | Yes / No / Unclear |
| Did patients receive the same reference standard?  *Did the same ‘expert’ perform the assessment for reference standard? If diagnostic criteria were used were the same diagnostic criteria used throughout? Was there verification by a second ‘expert’ clinician in any/all cases?* | Yes / No / Unclear |
| Were all patients included in the analysis? | Yes / No / Unclear |
| Could the patient flow have introduced bias?  Risk: Low / High / Unclear | |
