# Supplementary material for: Identifying dementia cases with routinely collected health data: A systematic review
Source: Alzheimers Dement. 2018 Aug;14(8):1038–51. doi: 10.1016/j.jalz.2018.02.016 (PMC6105076; doi:10.1016/j.jalz.2018.02.016)
Supplement: Appendix C [file mmc3.docx]

**Appendix C. Codes used by studies included in the review with corresponding descriptions**

Where coding systems use >4 digits (i.e. ICD-9-CM) these have been shortened to be consistent with original ICD systems.

Note: this list describes all codes used by the included studies in this systematic review for reference and does not reflect the authors’ recommended code list.

| **Code** | **Description** |
| --- | --- |
| **ICD9** |  |
| 046.1 | Jakob-Creutzfeldt disease |
| 290 | Senile dementia, simple type |
| 290.0 | Senile dementia, uncomplicated |
| 290.1 | Presenile dementia |
| 290.2 | Senile dementia, depressed or paranoid type |
| 290.3 | Senile dementia with acute confusional state |
| 290.4 | Arteriosclerotic dementia |
| 290.8 | Other senile and presenile organic psychotic conditions |
| 290.9 | Unspecified senile and presenile organic psychotic conditions |
| 291.2 | Other alcoholic dementia |
| 291.8 | Other specified alcoholic psychoses |
| 292.8 | Other specified drug psychoses |
| 294 | Korsakov's psychosis or syndrome, nonalcoholic |
| 294.1 | dementia in conditions classified elsewhere |
| 294.8 | Other specified organic psychotic conditions (chronic) |
| 294.9 | Unspecified organic psychotic conditions (chronic) |
| 331 | Alzheimer's disease |
| 331.1 | Pick's disease |
| 331.2 | Senile degeneration of brain |
| 331.7 | Cerebral degeneration in other diseases classified elsewhere |
| 331.8 | Other specified cerebral degeneration |
| 331.9 | Cerebral degeneration, unspecified |
| 332 | Paralysis agitans |
| 332.1 | Secondary parkinsonism |
| 333.4 | Huntington's chorea |
| 437.8 | Other ill-defined cerebrovascular disease |
| 438.0 | Late effects of cerebrovascular disease, cognitive deficits |
| 780.9 | Other general symptoms |
| 797 | Senility without mention of psychosis |
|  |  |
| **ICD 10** |  |
| A81.0 | Creutzfeldt-Jakob disease |
| E51.2 | Wernicke encephalopathy |
| F00 | Dementia in Alzheimer disease |
| F00.0 | Dementia in Alzheimer disease with early onset |
| F00.1 | Dementia in Alzheimer disease with late onset |
| F00.2 | Dementia in Alzheimer disease, atypical or mixed type |
| F00.9 | Dementia in Alzheimer disease, unspecified |
| F01 | Vascular dementia |
| F01.0 | Vascular dementia of acute onset |
| F01.1 | Multi-infarct dementia |
| F01.2 | Subcortical vascular dementia |
| F01.3 | Mixed cortical and subcortical vascular dementia |
| F01.8 | Other vascular dementia |
| F01.9 | Vascular dementia, unspecified |
| F02 | Dementia in other diseases classified elsewhere |
| F02.0 | Dementia in Pick disease |
| F02.1 | Dementia in Creutzfeldt-Jakob disease |
| F02.2 | Dementia in Huntington disease |
| F02.3 | Dementia in Parkinson disease |
| F02.4 | Dementia in human immunodeficiency virus [HIV] disease |
| F02.8 | Dementia in other specified diseases classified elsewhere |
| F03 | Unspecified dementia |
| F04 | Organic amnesic syndrome, not induced by alcohol or other psychoactive substances |
| F05.1 | Delirium superimposed on dementia |
| F10.6 | Mental and behavioural disorders due to use of alcohol, amnesic syndrome |
| F10.7 | Mental and behavioural disorders due to use of alcohol, residual and late-onset psychotic disorder |
| F16.7 | Mental and behavioural disorders due to use of hallucinogens |
| F18.7 | Mental and behavioural disorders due to use of volatile solvents |
| F19.7 | Mental and behavioural disorders due to multiple drug use and use of other psychoactive substances |
| G30 | Alzheimer disease |
| G30.0 | Alzheimer disease with early onset |
| G30.1 | Alzheimer disease with late onset |
| G30.8 | Other Alzheimer disease |
| G30.9 | Alzheimer disease, unspecified |
| G31.0 | Circumscribed brain atrophy |
| G31.1 | Senile degeneration of brain, not elsewhere classified |
| G31.8 | Other specified degenerative diseases of nervous system |
| G31.9 | Degenerative disease of nervous system, unspecified |
| I69.9 | Sequelae of cerebrovascular disease |
| R41 | Other symptoms and signs involving cognitive functions and awareness |
| R54 | Senility |
|  |  |
| **Read version 2** | |
| 66h.. | Dementia monitoring |
| 6AB.. | Dementia annual review |
| E00.. | Senile and presenile organic psychotic conditions |
| E000. | Uncomplicated senile dementia |
| E0010 | Uncomplicated presenile dementia |
| E0011 | Presenile dementia with delirium |
| E0012 | Presenile dementia with paranoia |
| E0013 | Presenile dementia with depression |
| E001z | Presenile dementia NOS |
| E002. | Senile dementia with depressive or paranoid features |
| E0020 | Senile dementia with paranoia |
| E0021 | Senile dementia with depression |
| E002z | Senile dementia with depressive or paranoid features NOS |
| E003. | Senile dementia with delirium |
| E004. | Arteriosclerotic dementia |
| E0040 | Uncomplicated arteriosclerotic dementia |
| E0041 | Arteriosclerotic dementia with delirium |
| E0042 | Arteriosclerotic dementia with paranoia |
| E0043 | Arteriosclerotic dementia with depression |
| E004z | Arteriosclerotic dementia NOS |
| E00y. | Other senile and presenile organic psychoses |
| E00z. | Senile or presenile psychoses NOS |
| E041. | Dementia in conditions EC |
| Eu00. | [X]Dementia in Alzheimer's disease |
| Eu000 | [X]Dementia in Alzheimer's disease with early onset |
| Eu001 | [X]Dementia in Alzheimer's disease with late onset |
| Eu002 | [X]Dementia in Alzheimer's dis, atypical or mixed type |
| Eu01. | [X]Vascular dementia |
| Eu010 | [X]Vascular dementia of acute onset |
| Eu011 | [X]Multi-infarct dementia |
| Eu012 | [X]Subcortical vascular dementia |
| Eu013 | [X]Mixed cortical and subcortical vascular dementia |
| Eu01y | [X]Other vascular dementia |
| Eu01z | [X]Vascular dementia, unspecified |
| Eu02. | [X]Dementia in other diseases classified elsewhere |
| Eu020 | [X]Dementia in Pick's disease |
| Eu021 | [X]Dementia in Creutzfeldt-Jakob disease |
| Eu022 | [X]Dementia in Huntington's disease |
| Eu023 | [X]Dementia in Parkinson's disease |
| Eu024 | [X]Dementia in human immunodef virus [HIV] disease |
| Eu025 | [X]Lewy body dementia |
| Eu02y | [X]Dementia in other specified diseases classif elsewhere |
| Eu02z | [X] Unspecified dementia |
| F110. | Alzheimer's disease |
| F1100 | Alzheimer's disease with early onset |
| F1101 | Alzheimer's disease with late onset |
| F111. | Pick's disease |
| F112. | Senile degeneration of brain |
| F116. | Lewy body disease |
| Fyu30 | [X]Other Alzheimer's disease |
