# Supplementary material for: Identifying dementia cases with routinely collected health data: A systematic review
Source: Alzheimers Dement. 2018 Aug;14(8):1038–51. doi: 10.1016/j.jalz.2018.02.016 (PMC6105076; doi:10.1016/j.jalz.2018.02.016)
Supplement: Appendix D [file mmc4.docx]

**Appendix D. QUADS-2 results demonstrating risk of bias and applicability concerns of studies**

|  | Risk of bias | | | | | Applicability | | |
| --- | --- | --- | --- | --- | --- | --- | --- | --- |
| First author & year | Patient selection | Index test | Reference standard | | Flow & timing | Patient selection | Index test | Reference standard |
| Bender 2014 | **✗** | **?** | | **✓** | **?** | **✗** | **✓** | **✓** |
| Bjertness 1998 | **✗** | **✓** | | **✓** | **✓** | **✗** | **✓** | **✓** |
| Brown 2016 | ✔ | ✔ | | ✔ | **✗** | ✔ | ✔ | **?** |
| Butler 2012 | **✗** | **✗** | | **✓** | **✓** | **✗** | **✗** | **✓** |
| Dahl 2007 | **✓** | **✓** | | **✓** | **✓** | **✓** | **✓** | **✓** |
| Dunn 2005 | **?** | **?** | | **✓** | **✓** | **✓** | **✓** | **✓** |
| Feldman 2012 | **?** | **?** | | **?** | **?** | **?** | **✓** | **✓** |
| Fisher 1992 | **?** | **?** | | **✓** | **✓** | **?** | **✓** | **✓** |
| Fujiyoshi 2017 | ✔ | ✔ | | ✔ | ✔ | ✔ | ✔ | ✔ |
| Heath 2015 | **✗** | ✔ | | ✔ | ✔ | ✔ | ✔ | ✔ |
| Henderson 2006 | **?** | **✓** | | **✓** | **?** | **✓** | **?** | **✓** |
| Jaakkimainen 2016 | ✔ | ✔ | | ✔ | ✔ | ✔ | ✔ | ✔ |
| Jin 2004 | **?** | **✓** | | **✓** | **✓** | **✓** | **✓** | **✓** |
| Juurlink 2006 | **?** | **?** | | **?** | **✓** | **✓** | **?** | **?** |
| Newens 1993 | **?** | **✓** | | **✓** | **✓** | **?** | **✓** | **✓** |
| Nielsen 2011 | **✗** | **✓** | | **✓** | **?** | **✗** | **✓** | **✓** |
| Ostbye 1999 | **✓** | **?** | | **✓** | **✓** | **✓** | **✓** | **✓** |
| Phung 2007 | **✓** | **✓** | | **✓** | **✓** | **✓** | **✓** | **✓** |
| Pippenger 2001 | **?** | **?** | | **✓** | **✓** | **✓** | **✓** | **✓** |
| Preen 2004 | **?** | **?** | | **✓** | **✓** | **✓** | **?** | **✓** |
| Quan 2008 | **?** | **?** | | **✓** | **✓** | **✓** | **✓** | **✓** |
| Romero 2014 | **✓** | **?** | | **?** | **✓** | **✓** | **✓** | **✓** |
| Salem 2012 | **✗** | **✓** | | **✓** | **✓** | **?** | **✓** | **✓** |
| Solomon 2014 | **✓** | **?** | | **✓** | **✓** | **✓** | **✓** | **✓** |
| Taylor 2009 | **✓** | **?** | | **✓** | **?** | **✓** | **✓** | **✓** |
| Van de Vorst 2015 | **✓** | **✓** | | **✓** | **✓** | **✓** | **✓** | **✓** |
| Wei 2016 | **✗** | **?** | | ✔ | ✔ | ✔ | **?** | ✔ |

✔ Low Risk **?** Unclear Risk ✘ High Risk
