# Supplementary material for: Identifying dementia cases with routinely collected health data: A systematic review
Source: Alzheimers Dement. 2018 Aug;14(8):1038–51. doi: 10.1016/j.jalz.2018.02.016 (PMC6105076; doi:10.1016/j.jalz.2018.02.016)
Supplement: Appendix E [file mmc5.docx]

**
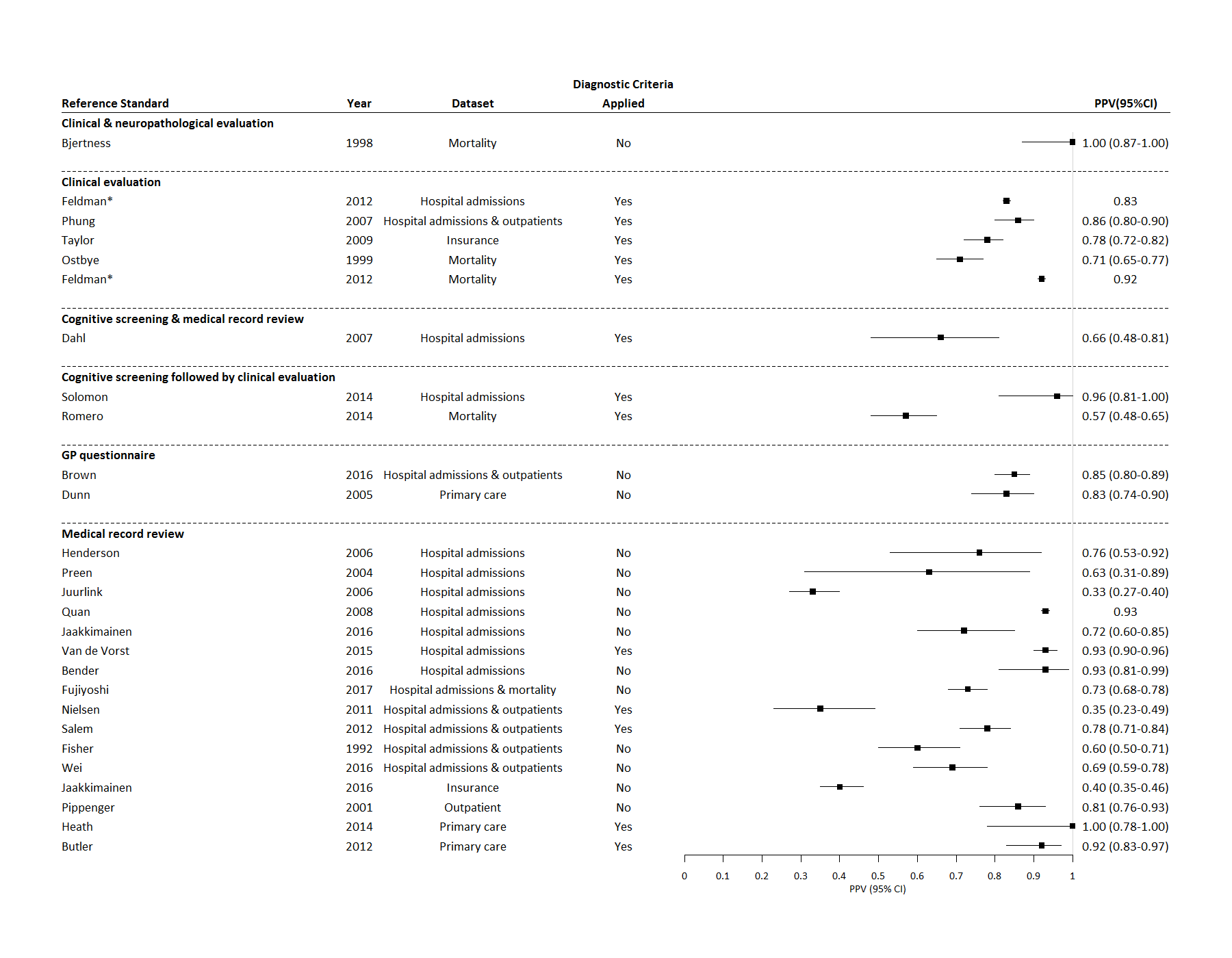
Appendix E. PPV estimates for all-cause dementia, stratified by reference standard**

**PPV estimates for routinely-collected coded health data to identify all-cause dementia cases, stratified by method of reference standard used in validation study.**

PPV: positive predictive value. Study size: number of cases with ≥1 dementia codes in dataset.

*High risk of bias or applicability concerns in one or more areas
