# Supplementary material for: Identifying dementia cases with routinely collected health data: A systematic review
Source: Alzheimers Dement. 2018 Aug;14(8):1038–51. doi: 10.1016/j.jalz.2018.02.016 (PMC6105076; doi:10.1016/j.jalz.2018.02.016)
Supplement: Appendix F [file mmc6.docx]

|  |  |  |  | **Positive predictive value** | | | | **Sensitivity** | | | |  |
| --- | --- | --- | --- | --- | --- | --- | --- | --- | --- | --- | --- | --- |
| **(A) Coding position** |  |  |  |  | | | |  | | | |  |
| Study | Country | Disorder | Dataset | Primary | | Any | | Primary | | Any | |  |
|  |  |  |  | TP/(TP+FP) | PPV % (95% CI) | TP/(TP+FP) | PPV % (95% CI) | TP/(TP+FN) | Sensitivity %  (95% CI) | TP/(TP+FN) | Sensitivity %  (95% CI) |  |
| Fisher 1992 | USA | Dem | H† | 13/17 | 76 (50-93) | 55/91 | 60 (50-69) | - | - | - | - |  |
| Newens 1993 | UK | Dem | D | - | - | - | - | 113/257 | 44 (38-50) | 167/257 | 65 (20-31) |  |
| Newens 1993 | UK | AD | D | - | - | - | - | 32/173 | 18 (13-25) | 49/173 | 28 (22-36) |  |
| Ostbye 1999 | Canada | Dem | D | 56/73 | 77 (65-86) | 170/240 | 71 (65-77) | 56/452 | 12 (10-16) | 170/452 | 38 (35-42) |  |
|  |  |  |  |  |  |  |  |  |  |  |  |  |
| **(B) Subtype code to indicate dementia** | | |  |  | | | |  | | | |  |
| Study | Country | Disorder | Dataset | Subtype code | | Any dementia code | | AD code | | Any dementia code | |  |
|  |  |  |  | TP/(TP+FP) | PPV % (95% CI) | TP/(TP+FP) | PPV % (95% CI) | TP/(TP+FN) | Sensitivity % (95% CI) | TP/(TP+FN) | Sensitivity % (95% CI) |  |
| Ostbye 1999 | Canada | AD | D | 68/79 | 86 (76-93) | 170/240 | 71 (65-77) | - | - | - | - |  |
| Phung 2007 | Denmark | AD&VD | H† | 55/58 | 94 (87-98) | 169/197 | 86 (80-90) | - | - | - | - |  |
|  |  |  |  |  |  |  |  |  |  |  |  |  |
| **(C) Patient age** |  |  |  |  | | | |  | | | |  |
| Study | Country | Disorder | Dataset | <65 years | | ≥65 years | | <65 years | | ≥65 years | |  |
|  |  |  |  | TP/(TP+FP) | PPV % (95% CI) | TP/(TP+FP) | PPV % (95% CI) | TP/(TP+FN) | Sensitivity % (95% CI) | TP/(TP+FN) | Sensitivity % (95% CI) |  |
| Van der Vorst 2015 | Netherlands | Dem | H | 19/28 | 68 (49-82) | 298/312 | 96 (93-97) | - | - | - | - |  |
|  |  |  |  |  |  |  |  |  |  |  |  |  |
| **(D) Dementia severity** | |  |  |  | | | |  | | | |  |
| Study | Country | Disorder | Dataset | Mild | | Moderate/severe | | Mild | | Moderate/severe | |  |
|  |  |  |  | TP/(TP+FP) | PPV % (95% CI) | TP/(TP+FP) | PPV % (95% CI) | TP/(TP+FN) | Sensitivity % (95% CI) | TP/(TP+FN) | Sensitivity % (95% CI) |  |
| Romero 2014 | Spain | Dem | D | - | - | - | - | 17/128 | 13 (8-20) | 54/222 | 24 (19-31) |  |
|  |  |  |  |  |  |  |  |  |  |  |  |  |
| **(E) Number of times dementia code appears in dataset** | | | |  |  |  |  |  |  |  |  |  |
| Study | Country | Disorder | Dataset | Once | | Twice or more | | Once | | Twice or more | |  |
|  |  |  |  | TP/(TP+FP) | PPV % (95% CI) | TP/(TP+FP) | PPV % (95% CI) | TP/(TP+FN) | Sensitivity % (95% CI) | TP/(TP+FN) | Sensitivity % (95% CI) |  |
| Brown 2016 | UK | Dem | H | 56/82 | 68 (57-78) | 152/162 | 94 (89-97) | - | - | - | - |  |
|  |  |  |  |  |  |  |  |  |  |  |  |  |
| **Results of within-study analyses, investigating the effects of (A) coding position, (B) using subtype codes to indicate all-cause dementia, (C) patient age and (D) severity of dementia on PPV and sensitivity**  PPV and sensitivity results calculated from raw figures, and 95% confidence intervals calculated using Clopper-Pearson exact method. Dem – dementia, AD – Alzheimer’s disease, VD – vascular dementia, TP – true positives, FP – false positives, FN – false negatives. H - hospital admissions data, H† - hospital admissions data from insurance dataset, D - death data | | | | | | | | | | | | |

**Appendix F. Results of within-study analyses**
